# Neurodegeneration in systemic lupus erythematosus: layer by layer retinal study using optical coherence tomography

**DOI:** 10.1186/s40942-020-00219-y

**Published:** 2020-04-21

**Authors:** Arnaldo Dias-Santos, Joana Tavares Ferreira, Sofia Pinheiro, João Paulo Cunha, Marta Alves, Ana L. Papoila, Maria Francisca Moraes-Fontes, Rui Proença

**Affiliations:** 1 Department of Ophthalmology, Centro Hospitalar Universitário de Lisboa Central, Lisbon, Portugal; 2 Department of Ophthalmology, Hospital CUF Descobertas, Lisbon, Portugal; 3 NOVA Medical School, Universidade NOVA de Lisboa, Lisbon, Portugal; 4 Autoimmune Disease Unit, Unidade de Doenças Auto-imunes/Serviço Medicina 3, Hospital de Santo António dos Capuchos, Centro Hospitalar Universitário de Lisboa Central, Lisbon, Portugal; 5 Epidemiology and Statistics Unit, Research Center, Centro Hospitalar Universitário de Lisboa Central, Lisbon, Portugal; 6 CEAUL (Center of Statistics and its Applications), Lisbon University, Lisbon, Portugal; 7 Autoimmune Disease Unit, Unidade de Doenças Auto-imunes/Serviço de Medicina 7.2, Hospital Curry Cabral, Centro Hospitalar Universitário de Lisboa Central, Lisbon, Portugal; 8 Instituto Gulbenkian de Ciência, Oeiras, Portugal; 9 Department of Ophthalmology, Centro Hospitalar Universitário de Coimbra, Coimbra, Portugal; 10 Faculty of Medicine, University of Coimbra, Coimbra, Portugal; 11 Serviço de Oftalmologia, Hospital de Santo António dos Capuchos, Alameda de Santo António dos Capuchos, 1169-050 Lisbon, Portugal

**Keywords:** Neurodegeneration, Peripapillary retinal nerve fiber layer, Photoreceptors, Spectral domain optical coherence tomography, Systemic lupus erythematosus

## Abstract

**Background:**

Systemic lupus erythematosus (SLE) is a chronic, autoimmune and multisystemic disease. Recent studies with functional and structural magnetic resonance imaging and cognitive tests report an unexpectedly high frequency of central nervous system involvement, even in patients with asymptomatic SLE. The purpose of this study was to identify early signs of retinal neurodegeneration by comparing the thickness of the peripapillary retinal nerve fiber layer (pRNFL) and all macular layers between patients with SLE without ophthalmologic manifestations and healthy controls. The effect of disease duration and systemic comorbidities was also studied.

**Methods:**

Cross-sectional study, in which all participants underwent a complete ophthalmologic evaluation including retinal segmentation analysis with spectral domain-optical coherence tomography. Patients with SLE also received a detailed autoimmune disease specialist evaluation to assess the disease activity state and systemic involvement. For pRNFL thickness, the global and six peripapillary sectors were determined. Each macular layer thickness was determined in the nine Early Treatment Diabetic Retinopathy Study (ETDRS) subfields. A multiple linear regression analysis was performed to control for the effect of potential demographic, ophthalmic and systemic confounders. A second multivariable analysis, including patients with SLE only, was performed to assess the effect of disease-specific variables on the outcome measures.

**Results:**

Sixty-eight eyes of 68 patients with SLE and 50 eyes of 50 healthy controls were considered. The pRNFL was significantly thinner in the SLE group globally (p = 0.026) and in the temporal superior (p = 0.007) and temporal (p = 0.037) sectors. In patients with SLE, chronic medication for hypercholesterolemia, hypertension and anticoagulants were associated with a significant thinning of the pRNFL. Patients with SLE presented significant thinning in the photoreceptor layer in five ETDRS areas (p < 0.05). Shorter disease duration was associated with greater photoreceptor thinning in all ETDRS subfields. Neuropsychiatric SLE, higher disease activity and cardiovascular risk factors were associated with a thinner photoreceptor layer. No differences were observed in overall retinal thickness or the remaining macular layers.

**Conclusion:**

Patients with SLE present early signs of retinal neurodegeneration, as evidenced by a reduction in the photoreceptor layer and pRNFL. These signs are more pronounced in patients with higher cardiovascular risk burden or neuropsychiatric involvement.

## Background

Systemic lupus erythematosus (SLE) is a chronic autoimmune disease that can affect multiple organ systems. Involvement of the central nervous system (CNS) occurs in 12% to 95% of patients with SLE [1]. A large number of manifestations ranging from distinct neurologic disorders such as stroke, aseptic meningitis and Guillain-Barré syndrome to more subtle dysfunctions such as headache, mood disorders and cognitive dysfunction have been included by the American College of Rheumatology under the designation of neuropsychiatric systemic lupus erythematosus (NP-SLE) [2]. Overall, it is associated with a significant increase in morbidity and mortality [3]. However, the absence of NP-SLE biomarkers and a lack of specificity for neuropsychiatric events that are common in the general population render the diagnosis a challenge. Moreover, the frequency of some sort of neurologic impairment in SLE is unexpectedly high, even in asymptomatic patients without NP-SLE criteria [4]. Of note, patients with non-NP-SLE perform worse in cognitive tests and present a significantly higher rate of structural and functional abnormalities in magnetic resonance imaging when compared to healthy controls [5]. Additionally, the brains of recently diagnosed patients with SLE, without neurologic symptoms, present abnormally high blood flow and glucose consumption (hypermetabolism) in the white matter. This phenomenon was more prominent in patients with poor systemic disease control [6]. This finding suggests that neurologic involvement with CNS inflammation and subsequent neurodegeneration may be present early in the course of the disease before the development of NP-SLE.

Spectral domain optical coherence tomography (SD-OCT) is a safe and objective method that permits high-resolution cross-sectional images of the retina. In the last few years, this technique has evolved, allowing precise qualitative and quantitative evaluation of all retinal layers with high repeatability and reproducibility [7]. This evolution expanded its applications beyond ophthalmic diseases, and SD-OCT is now a well-established biomarker of neurodegenerative disorders such as multiple sclerosis [8], Alzheimer’s disease [9] and Parkinson’s disease [10]. The role of OCT as a biomarker of SLE-associated neurodegeneration has been addressed only in a few studies, which have yielded inconsistent results. However, these were pilot studies with small sample sizes, which only evaluated total retinal thickness or focused on some retinal layers [11–13].

This study aimed to compare the thickness of all macular layers as well as the peripapillary retinal nerve fiber layer (pRNFL) thickness between patients with SLE without ophthalmologic manifestations and a healthy control group. The relationship between retinal thickness and demographic characteristics, as well as disease duration, hydroxychloroquine intake and cumulative dosage and systemic comorbidities such as neuropsychiatric SLE, lupus nephritis and antiphospholipid syndrome was also studied.

## Methods

### Patients

This was a cross-sectional study performed at the Ophthalmology Department and at the Autoimmune Disease Units of the Central Lisbon Hospital University Center between August 2017 and August 2018. Consecutive patients with SLE were screened for inclusion/exclusion criteria. All patients fulfilled the 1997 revised American College of Rheumatology criteria for the diagnosis of SLE [14] and were aged between 18 and 80 years old.

A sex-matched control group with an age range between 18 and 80 years old was randomly recruited from the General Ophthalmology Department. Autoimmune diseases were ruled out in this group based on patient-reported past medical history and on general practitioner medical records.

The exclusion criteria for both study groups were a spherical equivalent > 5 diopters, axial length > 25 mm or keratic astigmatism > 3 diopters, diabetes mellitus, pregnancy, signs or previous history of optic neuropathy, retinopathy or choroidopathy (namely, lupus-related, age-related macular degeneration, vascular occlusion, macular dystrophy, hydroxychloroquine retinopathy, glaucoma, ocular hypertension or neurodegenerative diseases such as Alzheimer’s or Parkinson’s disease), ocular tumor, previous episodes of intraocular inflammation, history of intraocular or refractive surgery and substantial media opacities that compromised fundus imaging.

This study was approved by the Institutional Ethics Committee. All participants gave their written informed consent, and the principles of the Declaration of Helsinki were followed.

### Study procedures

A complete ophthalmologic evaluation was performed on all patients, where demographic, background medical history, full ophthalmological examination with best corrected visual acuity (BCVA) testing, slit-lamp biomicroscopy, dilated fundus examination, Goldmann applanation tonometry, optic biometry (using Lenstar LS 900^®^, Haag Streit AG, Koeniz, Switzerland) and SD-OCT were performed. Blood pressure was measured before SD-OCT. Patients with SLE currently or previously treated with hydroxychloroquine also underwent fundus autofluorescence imaging and 10-2 macular automated threshold visual field testing (using Octopus 900^®^, Haag Streit AG, Koeniz, Switzerland) to exclude retinal toxicity, in accordance with the American Academy of Ophthalmology guidelines [15]. An evaluation by an autoimmune disease specialist was also performed in all patients with SLE, which included a complete physical examination and laboratory tests required to access systemic disease activity, which was scored using the Systemic Lupus Erythematosus Disease Activity Index (SLEDAI) [16]. One eye per patient was randomly selected for the study.

### Spectral-domain optical coherence tomography imaging and layer segmentation

Peripapillary and macular tomographic scans were obtained with SD-OCT (Spectralis^®^ Heidelberg, software version 6.0 Heidelberg, Germany) in all participants after pupillary dilation. Only good quality scans, with a signal strength better than 20 (40 = maximum), with completely focused images, without artifacts or blank areas were considered in the analysis.

**Fig. 1:**
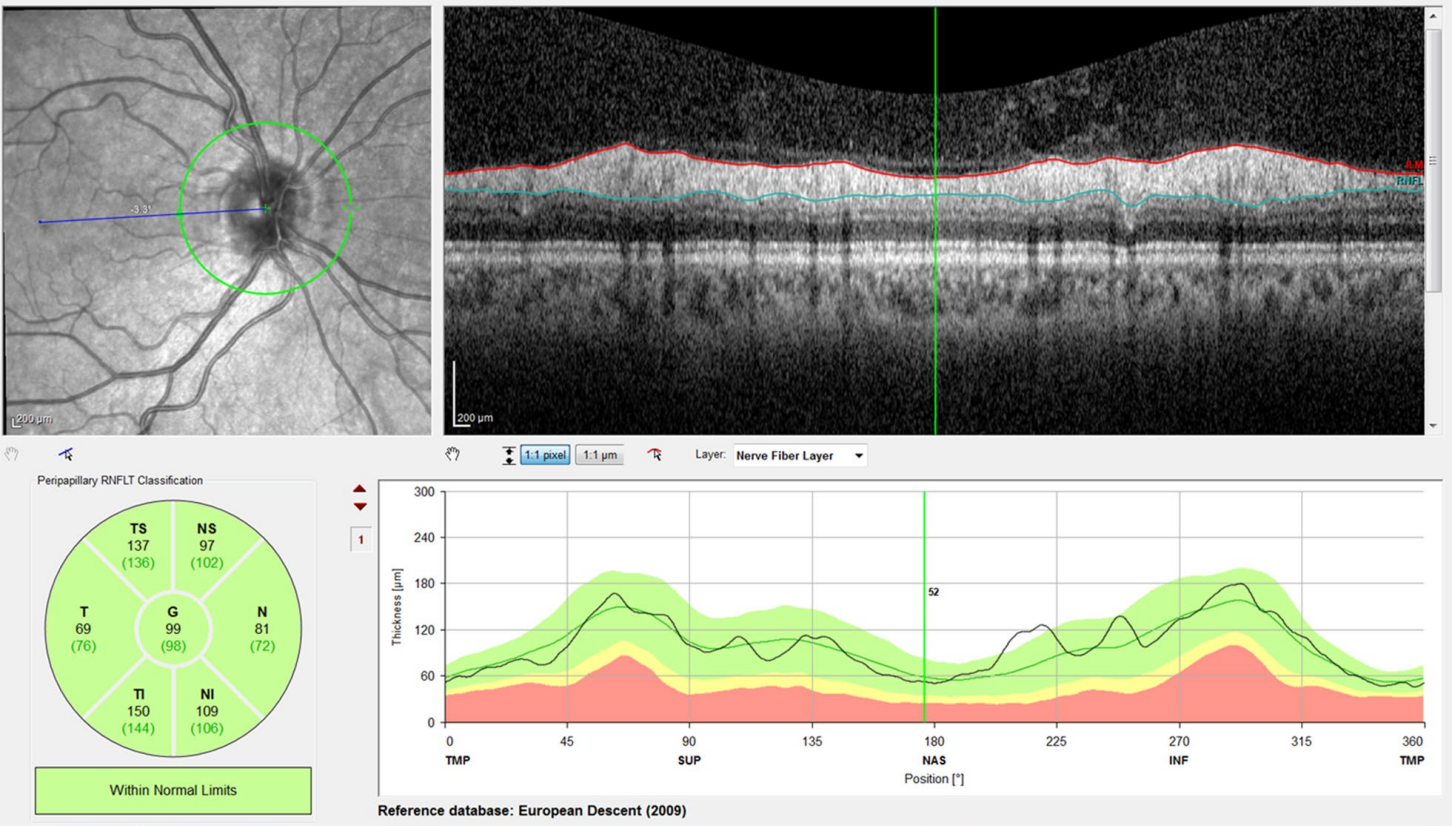
Thickness of the peripapillary retinal nerve fiber layer obtained by “RNFL Single Exam Report OU with FoDi™” (Spectralis Heidelberg; μm)

The pRNFL measurements employed a 12° circular scan centered on the optic nerve head, which corresponds to a retinal diameter of approximately 3.46 mm in an eye with typical axial length and corneal curvature. pRNFL thickness was determined globally (G) and for the six peripapillary sectors (TS—temporal superior, T—temporal, TI—temporal inferior, NI—nasal inferior, N—nasal, and NS—nasal superior) by the instrument’s built-in software, which automatically segments the internal limiting membrane (ILM) and the posterior border of the retinal nerve fiber layer (RNFL) (Fig. 1).

**Fig. 2:**
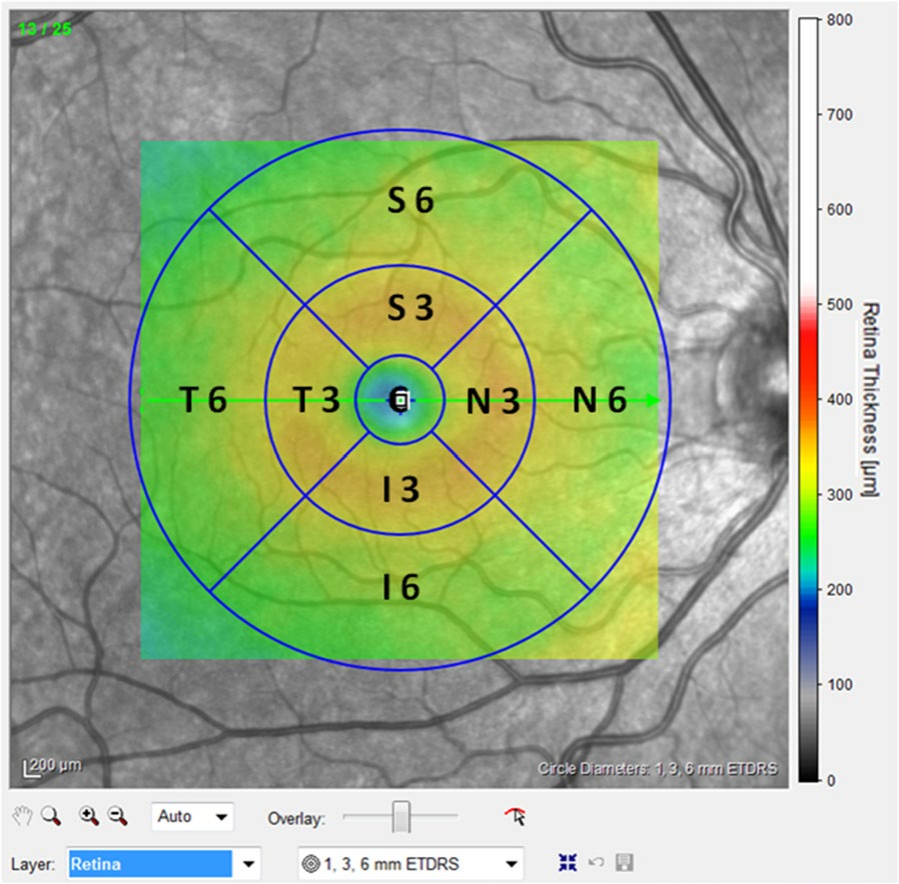
Representative Spectralis Heidelberg SD-OCT scans of the macular thickness map using the ETDRS protocol

For macular measurements, a fast macular thickness OCT protocol was used, which consists of horizontal raster scans obtained in a 20 × 20° (5.8 mm × 5.8 mm) square centered on the fovea (25 high-resolution scans with nine frames per B-scan). Images were analyzed using Spectralis automatic segmentation software to calculate individual retinal layer thickness values, namely, overall retinal thickness (RT), RNFL, ganglion cell layer (GCL), inner plexiform layer (IPL), inner nuclear layer (INL), outer plexiform layer (OPL), outer nuclear layer (ONL), photoreceptor layer (PRL) and retinal pigment epithelium (RPE). An Early Treatment Diabetic Retinopathy Study (ETDRS) plot was automatically projected onto the retina by Spectralis OCT software [17]. This plot consists of three concentric rings centered on the fovea, with 1-, 3- and 6-mm diameters. Two intersecting lines divide the two outer ETDRS rings into quadrants, generating nine sectors: C, S3, T3, I3, N3, S6, T6, I6 and N6 (Fig. 2). Mean thickness values for all retinal layers were recorded for the nine ETDRS sectors.

Enhanced depth imaging scans were also obtained using the fast macular thickness OCT protocol to measure choroidal thickness (CT), according to a previously described method [18]. CT was measured manually, subfoveally and at 1000 µm superior, temporal, inferior and nasal to the fovea.

All OCT examinations were performed between 2:00 PM and 4:00 PM by an ophthalmologist (J.T.F.) and were assessed by another ophthalmologist (A.D.S.), both masked to the patients’ diagnosis. Image quality, centration and segmentation were checked and corrected if necessary.

### Statistical analysis

The characteristics of study participants were described using the mean (standard deviation: SD) or the median (interquartile range: P_25_–P_75_) for continuous variables and frequencies (percentages) for categorical variables.

Univariable and multivariable linear regression models were used to identify the variables that may explain the variability of macular retinal layers and pRNFL thicknesses. The effect of potential confounders such as age, sex, spherical equivalent, BCVA, axial length, choroidal thickness, intraocular pressure (IOP), mean arterial pressure (MAP), body mass index (BMI) and systemic medication was taken into account in this analysis. The selection of variables for the multivariable analysis was performed using the backward elimination method.

An analysis only for the SLE group was also performed to study the effect on retinal thickness of disease duration, hydroxychloroquine intake and cumulative dosage and systemic comorbidities such as neuropsychiatric SLE, lupus nephritis and antiphospholipid syndrome, among others, using the same regression models.

The normality assumption of the residuals was verified with the Shapiro–Wilk goodness-of-fit test. A level of significance of α = 0.05 was considered. Data were analyzed using the Statistical Package for the Social Sciences for Windows, version 22.0 (IBM Corp. Released 2013. IBM SPSS Statistics for Windows, Version 22.0; IBM Corp, Armonk, NY).

## Results

### Patient demographics and clinical characteristics

A total of 68 eyes of 68 patients with SLE (58 women and 10 men) and 50 eyes of 50 healthy controls (43 women and 7 men) were enrolled in this study. The clinical and demographic characteristics of the patients and controls are summarized in Table 1. The pharmacological history is summarized in Additional file 1.

**Table 1:** Demographic and clinical characteristics of the patients by group

Variables	SLE group (n = 68)	Control group (n = 50)	p-value
Age, years	45.50 (12.67)	52.76 (14.45)	0.003
Female sex, n (%)	58 (85.3)	43 (86)	0.914*
Body mass index, kg/m^2^	24.64 (3.91)	25.79 (3.73)	0.070
BCVA, logMAR	0.010 (0.051)	0.005 (0.020)	0.890
IOP-Goldmann, mm Hg	13.60 (2.88)	13.76 (2.55)	0.738
Spherical equivalent, D	− 0.25 (− 1.0 to 0.25)	0.13 (− 0.63 to 1.0)	0.048
Axial length, mm	23.56 (1.00)	22.89 (0.96)	< 0.001
MAP, mm Hg	88.71 (11.06)	91.92 (13.11)	0.114
SLE duration, years	11.0 (6.25–19.0)	NA	
SLEDAI	2 (0–4)	NA	
HCQ			
Daily dose, mg	329 (96)	NA	
Cumulative dose, g	778 (228.1–1606.0)	NA	
Therapy duration, years	5.30 (1.81–11.83)	NA	
Daily dose/weight, mg/kg	5.02 (1.61)	NA	
Cumulative dose/weight, g/kg	10.76 (3.16–25.47)	NA	
NP-SLE, n (%)	19 (27.9)	NA	
Central NP-SLE, n (%)	16 (23.5)	NA	
Peripheral NP-SLE, n (%)	3 (4.4)	NA	
Lupus nephritis, n (%)	18 (26.5)	NA	
Antiphospholipid syndrome, n (%)	21 (30.9)	NA	
Sjogren’s syndrome, n (%)	5 (7.4)	NA	

The results are expressed as the mean (SD) or median (P_25_–P_75_) for continuous variables or as n (%) for categorical variables

*BCVA* best corrected visual acuity, *HCQ* hydroxychloroquine, *IOP* intraocular pressure, *logMAR* logarithm of the minimum angle of resolution, *MAP* mean arterial pressure, *NA* not applicable, *NP-SLE* neuropsychiatric systemic lupus erythematosus, *SLE* systemic lupus erythematosus, *SLEDAI* Systemic Lupus Erythematosus Disease Activity Index

*Chi-square test; remaining p-values were obtained by the Mann–Whitney test

### Analysis of the peripapillary retinal nerve fiber layer

Univariable analysis revealed a statistically significant difference in pRNFL thickness in the TS and T sectors, which was lower in the SLE group (Additional file 2).

In multivariable linear regression models (Table 2), after adjusting for sex, age, BCVA, IOP, spherical equivalent, axial length, BMI, MAP, and systemic medication, there was a statistically significant difference in pRNFL thickness for the global (p = 0.026), temporal superior (p = 0.007) and temporal sectors (p = 0.037), which was lower in the SLE group. Age was negatively associated with pRNFL in some sectors (G p < 0.001; TI p = 0.013; NS p = 0.013; N p = 0.025). Axial length was also negatively associated with pRNFL thickness in the G (p = 0.013) and NI (p = 0.007) sectors.

**Table 2:** Results of multivariable regression models—dependent variable: pRNFL thickness

Model	Coefficient estimate	95% confidence interval	p-value
Dependent variable: pRNFL thickness G			
SLE group*	− 3.87	− 7.27 to − 0.48	0.026
Male sex	− 4.93	− 9.39 to − 0.46	0.031
Age (years)	− 2.60	− 3.87 to − 1.32	< 0.001
Axial length (mm)	− 2.23	− 3.97 to − 0.49	0.013
Benzodiazepines	7.37	1.70 to 13.04	0.011
Dependent variable: pRNFL thickness TS			
SLE group*	− 7.94	− 13.64 to − 2.38	0.007
Dependent variable: pRNFL thickness T			
SLE group*	− 4.14	− 8.03 to − 0.25	0.037
Dependent variable: pRNFL thickness TI			
SLE group*	− 3.95	− 11.17 to 3.28	0.282
Age (years)	− 3.28	− 5.86 to − 0.69	0.013
Dependent variable: pRNFL thickness NI			
SLE group*	6.39	− 2.59 to 15.36	0.161
Axial length (mm)	− 5.91	− 10.21 to − 1.61	0.007
Dependent variable: pRNFL thickness N			
SLE group*	− 2.42	− 6.94 to 2.10	0.291
Age (years)	− 1.86	− 3.47 to − 0.24	0.025
Dependent variable: pRNFL thickness NS			
SLE group*	− 7.04	− 14.53 to 0.46	0.065
Age (years)	− 3.40	− 6.08 to − 0.72	0.013

*Reference category: control group. Age: for each 10-year increase. p-values were obtained by linear regression models

*G* global, *N* nasal, *NI* nasal inferior, *NS* nasal superior, *pRNFL* peripapillary retinal nerve fiber layer, *SLE* systemic lupus erythematosus, *T* temporal, *TI* temporal inferior, *TS* temporal superior

The results of the multivariable regression analysis considering the SLE group alone are summarized in Table 3. Notably, chronic medication with antihypertensive drugs, namely, calcium channel blockers or angiotensin-converting enzyme inhibitors, was associated with a thinner pRNFL in some sectors (G p = 0.009; TI p = 0.017; N p = 0.023; NS p = 0.006). Chronic medication with anticoagulants was negatively associated with pRNFL thickness in the G (p = 0.022), TI (p = 0.007), NI (p = 0.020) and NS (p = 0.037) sectors. Chronic medication with statins was also negatively associated with pRNFL thickness in two sectors: TI (p = 0.021) and NI (p = 0.020). Age and axial length also remained in the model in some pRNFL sectors.

**Table 3:** Results of multivariable regression models for the SLE group—dependent variable: pRNFL thickness

Model	Coefficient estimate	95% confidence interval	p-value
Dependent variable: pRNFL thickness G			
Age (years)	− 3.02	− 4.91 to − 1.13	0.002
Axial length (mm)	− 3.98	− 6.34 to − 1.62	0.001
Calcium channel blocker	− 10.66	− 18.56 to − 2.77	0.009
Anticoagulant	− 6.71	− 12.42 to − 1.00	0.022
Dependent variable: pRNFL thickness TS			
Age (years)	− 3.38	− 6.55 to − 0.22	0.037
Axial length (mm)	− 4.12	− 8.13 to − 0.12	0.044
Dependent variable: pRNFL thickness T			
Sjogren syndrome	− 10.36	− 19.38 to − 1.35	0.025
Dependent variable: pRNFL thickness TI			
IOP (mm Hg)	− 2.15	− 3.60 to − 0.71	0.004
Calcium channel blocker	− 17.97	− 32.64 to − 3.30	0.017
Anticoagulant	− 15.08	− 25.84 to − 4.31	0.007
Statin	− 13.94	− 25.73 to − 2.15	0.021
Dependent variable: pRNFL thickness NI			
Axial length (mm)	− 9.03	− 14.59 to − 3.47	0.002
Anticoagulant	− 17.16	− 31.56 to − 2.76	0.020
Statin	− 18.56	− 34.09 to − 3.03	0.020
Dependent variable: pRNFL thickness N			
Body mass index (kg/m^2^)	− 0.80	− 1.58 to − 0.01	0.046
ACE inhibitor	− 10.03	− 18.61 to − 1.45	0.023
Dependent variable: pRNFL thickness NS			
Axial length (mm)	− 5.47	− 9.46 to − 1.47	0.008
Calcium channel blocker	− 20.07	− 34.01 to − 6.12	0.006
Anticoagulant	− 11.34	− 21.99 to − 0.69	0.037
Corticosteroid (mg)	0.12	0.05 to 0.18	0.001

Age: for each 10-year increase. Corticosteroid is expressed as the daily dose of prednisone equivalent. p-values were obtained by linear regression models

*ACE* angiotensin-converting enzyme, *G* global, *IOP* intraocular pressure, *N* nasal, *NI* nasal inferior, *NS* nasal superior, *pRNFL* peripapillary retinal nerve fiber layer, *SLE* systemic lupus erythematosus, *T* temporal, *TI* temporal inferior, *TS* temporal superior

### Analysis of macular retinal layers thickness

**Fig. 3:**
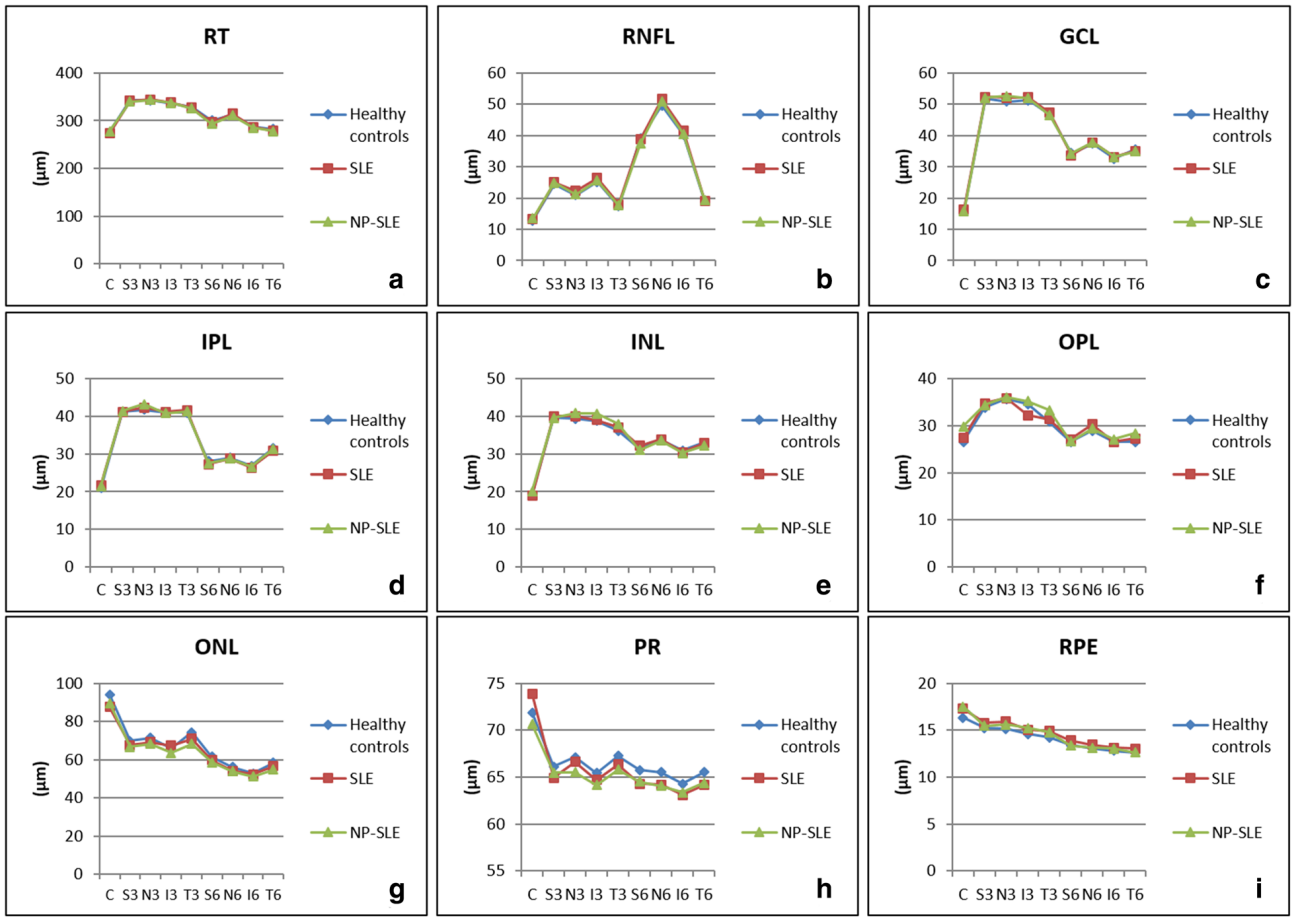
Graphs displaying individual retinal layer thickness in the nine ETDRS areas for the SLE group, the control group and the neuropsychiatric SLE subgroup. **a** RT; **b** RNFL; **c** GCL; **d** IPL; **e** INL; **f** OPL; **g** ONL; **h** PRL; **i** RPE

The mean retinal layers thickness in the healthy control and SLE groups, as well as in the subgroup of patients with NP-SLE, is depicted in Fig. 3.

In the multivariable analysis, after considering age, sex, BCVA, IOP, spherical equivalent, axial length, BMI, MAP and systemic medication, there were no statistically significant differences in overall RT or in most locations for the remaining retinal layers. The only exception was the photoreceptor (PR) layer.

The results in the PR layer were more consistent and were thinner in the SLE group. Multivariable regression analysis was also performed by dividing the SLE group according to disease duration: group I (< 5 years, n = 16), group II (5–10 years, n = 17) and group III (> 10 years, n = 35). After classifying the SLE group according to this parameter, we observed a reduction in PR layer thickness when compared with the healthy control group that was attenuated with increasing disease duration (Table 4). This pattern of PR thinning occurs in all macular sectors.

**Table 4:** Results of multivariable regression models for PR layer thickness

Model*	Coefficient estimate	95% confidence interval	p-value
Dependent variable: PR layer thickness at S3 sector			
Group I	− 1.60	− 3.31 to 0.11	0.066
Group II	− 1.51	− 3.18 to 0.16	0.076
Group III	− 0.53	− 1.84 to 0.78	0.426
Dependent variable: PR layer thickness at T3 sector			
Group I	− 1.57	− 3.36 to 0.22	0.084
Group II	− 1.08	− 2.83 to 0.66	0.222
Group III	− 0.86	− 2.23 to 0.51	0.217
Dependent variable: PR layer thickness at I3 sector			
Group I	− 1.44	− 2.77 to − 0.11	0.035
Group II	− 0.79	− 2.10 to 0.51	0.231
Group III	− 0.70	− 1.72 to 0.33	0.180
Dependent variable: PR layer thickness at S6 sector			
Group I	− 1.74	− 3.05 to − 0.43	0.010
Group II	− 1.51	− 2.79 to − 0.22	0.022
Group III	− 1.20	− 2.21 to − 0.19	0.020
Dependent variable: PR layer thickness at T6 sector			
Group I	− 1.90	− 3.11 to − 0.68	0.003
Group II	− 1.52	− 2.71 to − 0.33	0.013
Group III	− 0.95	− 1.88 to − 0.02	0.046
Dependent variable: PR layer thickness at I6 sector			
Group I	− 1.24	− 2.32 to − 0.15	0.026
Group II	− 1.18	− 2.24 to − 0.12	0.029
Group III	− 1.04	− 1.88 to − 0.21	0.014
Dependent variable: PR layer thickness at N6 sector			
Group I	− 1.71	− 2.93 to − 0.49	0.007
Group II	− 1.40	− 2.60 to − 0.21	0.022
Group III	− 1.18	− 2.11 to − 0.24	0.014

*Reference category: healthy control group. p-values were obtained by linear regression models

*PR* photoreceptor, *Group I* up to 5 years of disease duration, *Group II* 5–10 years, *Group III* more than 10 years

A multivariable regression analysis for the PR layer was also performed, considering the SLE group alone (Table 5). A higher SLEDAI score was associated with a reduction in PR layer thickness in the C (p = 0.015) and N3 (p = 0.050) sectors. NP-SLE diagnosis was associated with thinning of the PR layer in the C sector (p = 0.025). Chronic medication for hypertension (calcium channel blockers and angiotensin-converting enzyme inhibitors) was associated with a thinner PR layer in the T3 (p = 0.039) and N6 (p = 0.043) sectors. Chronic medication with statins was also negatively associated with PR layer thickness in two sectors: I3 (p = 0.028) and N3 (p = 0.006). Finally, secondary Sjogren’s syndrome was associated with a thicker PR layer in the C (p = 0.015) and N3 (p = 0.048) sectors.

**Table 5:** Results of multivariable regression models for PR layer thickness in the SLE group

Model*	Coefficient estimate	95% confidence interval	p-value
Dependent variable: PR layer thickness at C sector			
NP-SLE	− 2.83	− 5.29 to − 0.37	0.025
Sjogren’s syndrome	5.05	0.71 to 9.40	0.023
SLEDAI	− 0.29	− 0.51 to − 0.06	0.015
Body mass index (kg/m^2^)	− 0.31	− 0.60 to − 0.02	0.038
Dependent variable: PR layer thickness at S3 sector			
Benzodiazepine	2.67	0.35 to 4.99	0.025
Dependent variable: PR layer thickness at T3 sector			
Calcium channel blocker	− 2.68	− 5.23 to − 0.14	0.039
Dependent variable: PR layer thickness at I3 sector			
Statin	− 1.84	− 3.48 to − 0.20	0.028
Dependent variable: PR layer thickness at N3 sector			
Sjogren’s syndrome	2.38	0.2 to 4.74	0.048
SLEDAI	− 0.13	− 0.26 to 0.00	0.050
Statin	− 2.44	− 4.15 to − 0.73	0.006
Dependent variable: PR layer thickness at S6 sector			
Benzodiazepine	1.95	0.23 to 3.68	0.027
HCQ treatment time (years)	− 0.83	− 0.16 to 0.00	0.041
Dependent variable: PR layer thickness at N6 sector			
Axial length (mm)	0.53	0.01 to 1.05	0.045
ACE inhibitor	− 1.50	− 2.95 to − 0.05	0.043

No multiple model was achieved for the T6 and I6 sectors. p-values were obtained by linear regression models

*ACE* angiotensin-converting enzyme, *HCQ* hydroxychloroquine, *NP-SLE* neuropsychiatric systemic lupus erythematosus, *PR* photoreceptor, *SLEDAI* Systemic Lupus Erythematosus Disease Activity Index

## Discussion

In this study, we found evidence of retinal neurodegeneration in patients with SLE. Using SD-OCT, we compared pRNFL and macular layers thickness between patients with SLE without ophthalmologic manifestations and healthy controls. A significant decrease in the pRNFL as well as in the macular photoreceptor layer was observed in patients with SLE.

It is believed that patients with SLE have chronic low-grade CNS inflammation beginning early in the course of the disease [19]. This inflammatory process profoundly impacts the continuous crosstalk held between the CNS and the immune system, resulting in the emergence of symptoms such as depression, anxiety disorders or psychosis [20]. Its pathophysiology is complex and multifactorial, involving immune complex depositions, autoantibody-mediated neuronal cell damage, inflammatory and thrombotic microangiopathy, intrathecal synthesis of proinflammatory cytokines and disruption of the blood–brain barrier [4]. This inflammatory microenvironment ultimately leads to mitochondrial damage and disturbance of neuron-glia metabolic coupling, resulting in a marked reduction in synaptic activity and neuronal death, which are the hallmarks of neurodegeneration [1, 4]. Indeed, the cerebrospinal fluid of patients with SLE presents increased levels of neurofilament (a neuronal degradation product), Tau (an axonal degeneration product) and astroglial fibrillary acidic protein (a marker of neuronal damage and gliosis) compared to that of healthy controls [21, 22].

Peripapillary RNFL thinning, specifically involving the temporal sectors, is a proven biomarker of neurodegeneration in several diseases, such as Alzheimer’s disease [9], Parkinson’s disease [10] or multiple sclerosis [8]. In this study, we documented a significant reduction in the pRNFL in the global, temporal superior and temporal sectors in patients with SLE compared to healthy controls. This thinning was independent of sex, age, BCVA, IOP, spherical equivalent, axial length, BMI, MAP and systemic medication. Age and axial length were also negatively associated with pRNFL thickness in some sectors. A multivariable analysis in the SLE group revealed that patients under chronic medication with anticoagulants, statins, angiotensin-converting enzyme inhibitors (ACEIs) and calcium channel blockers (CCBs) had thinner pRNFLs. Previous studies of pRNFL in SLE are scarce and had inconsistent results. Shulman et al. found a trend towards lower pRNFL thickness in SLE, without statistical significance. However, their sample was small, including only 21 patients with SLE and 11 healthy controls [12]. On the other hand, Liu et al. [11], in a study with 31 patients with SLE and 16 healthy controls, reported significant thinning in the global, temporal superior and nasal pRNFL. Additionally, they described a positive correlation between pRNFL thickness in the temporal superior and temporal inferior sectors and cognitive test scores. However, none of the abovementioned studies included a multivariable analysis; thus, the results for pRNFL thickness were not corrected for the effects of confounding factors such as age or axial length.

Macular layers analysis revealed a significant reduction in PR layer thickness in patients with SLE compared to healthy controls. Photoreceptors are the most energy-demanding neurons in the CNS [23]. Their metabolic demands are fulfilled by the choroidal circulation, particularly the choriocapillaris. Lupus choroidopathy has been extensively described and is characterized by choroidal vasculitis, as well as complement and immunoglobulin deposition in choroidal vessels, resulting in long-term choroidal ischemia [24]. Changes in choroidal circulation have also been described even in asymptomatic patients [25–27]. Vasculopathy of the choriocapillary layer may thus result in chronic ischemia of the outer retinal layers, particularly the PR, increasing reactive oxygen species production. This adverse environment reduces mitochondrial biogenesis, generating an energy crisis that causes the PR degeneration observed in patients with SLE [1]. Specific autoantibodies targeting photoreceptors are another proposed mechanism [28]. Destruction of the PR cell layer has been documented histologically in mouse models of SLE, along with thickening of the wall of the choroidal arterioles and occlusion of the choriocapillaries [29]. Ocular histopathologic reports on patients with lupus are rare in the literature. Cao et al. reported a case with diffuse loss of PR without significant signs of tissue inflammation. Additionally, the optic nerves presented loss of axons and thickened septae, with slight macrophage infiltration and microglial accumulation [28]. Thinning of the PR cell layer as an early sign of retinal neurodegeneration has already been described in other entities, such as diabetes mellitus and metabolic syndrome, in which microvascular disease assumes a central role in the pathogenesis [30, 31].

Considering patients with SLE only, the multivariable analysis of PR layer thickness revealed that patients with NP-SLE diagnosis had thinner values for the central sector. A higher disease activity index was also negatively associated with PR layer thickness in the central and N3 subfields. Patients with secondary Sjogren’s syndrome presented a thicker PR layer in the central and N3 sectors; however, this result must be interpreted with caution since there were only five patients with Sjogren’s syndrome. A thinner PR layer in some sectors of patients under chronic medication with antihypertensives (ACEIs and CCBs) and statins is also worth noting. This analysis suggests that patients with clinical neurological involvement as well as those with poor systemic disease control are more prone to PR degeneration. Similar to the pRNFL analysis, patients on chronic medication for hypertension or hypercholesterolemia are more susceptible to PR degeneration, suggesting a deleterious role of cardiovascular risk factors. A negative association between PR layer thickness and HCQ treatment time was observed in the S6 sector. However, the absence of significant associations in other ETDRS locations or with cumulative HCQ dosage, as well as the absence of characteristic visual field defects and fundus autofluorescence abnormalities, are arguments against the existence of HCQ macular toxicity.

Another interesting finding in this investigation was the pattern of PR layer thinning throughout the course of the disease. PR layer thinning is more marked in those patients with shorter disease duration (up to 5 years), followed by those with intermediate duration and then those with longer disease duration (more than 10 years). This pattern, which was observed in all ETDRS areas, may be interpreted as a result of retinal remodeling. Remodeling is a process in which the loss of cones and/or rods results in neural retina deafferentation, which in turn results in a series of changes to retinal organization [32]. Retinal remodeling has been described following retinal detachment [33], age-related macular degeneration [34] or any other situation where photoreceptors are lost, especially cones [32]. Regardless of the initiating event, the subsequent retinal changes lead to revisions in neuronal morphology and organization through neuritogenesis and cell migration, reorganization of synaptic connectivity and intracellular molecular processes [32]. This process of retinal plasticity may thus explain the partial recovery in PR layer thickness observed with longer disease duration.

Some studies evaluated subclinical retinal changes in other autoimmune systemic disorders using SD-OCT. A study in patients with rheumatoid arthritis revealed no significant changes in pRNFL or total foveal thickness compared to healthy controls [35]. On the other hand, patients with primary Sjogren’s syndrome present a significant reduction in pRNFL as well as in macular GCL compared to healthy controls [36]. Patients with Behçet’s disease also present significant thinning in the pRNFL [37]. Patients with neuro-Behçet present not only a reduction in pRNFL but also a reduction in total macular thickness compared to healthy controls [38]. Patients with Susac syndrome present a reduction in pRNFL, particularly in the nasal quadrant. These patients also present an abnormal foveal contour with a reduction in the thickness of the inner macular layers [39–41].

This study has some limitations. The first is the size of the sample. Despite being the study with the largest sample in this field, the sample size may have limited the ability to find differences in some subsets of patients, such as those with NP-SLE. However, significant thinning in the PR layer was observed in this subgroup in the central ETDRS subfield. Second, our assessment of disease duration is based on the time of the diagnosis, which, in some cases, may underestimate the real time of disease. Finally, retinal segmentation was performed by automatic software, which is susceptible to some mistakes. Regarding the pRNFL, decreased scan quality, tilted disc, older age and thinner pRNFL tend to underestimate automatic pRNFL thickness measurement. However, this inaccuracy can be significantly reduced by postsegmentation inspection and refinement by a trained operator [42, 43]. Regarding individual macular layer thickness measurements, Spectralis segmentation software has proven high repeatability and reproducibility for all layers in all nine ETDRS areas [7, 44]. Nevertheless, its accuracy may be significantly affected by image quality or marked anatomical distortion [7, 45]. In our study, we only included participants without retinal or optic nerve pathology in whom good quality scans could be obtained. Additionally, detailed inspection and manual refinement by an ophthalmologist masked to the patients’ diagnosis was performed whenever segmentation was deemed inaccurate.

## Conclusions

In conclusion, this study, which was the first to perform a complete SD-OCT segmentation analysis of all retinal layers in patients with SLE, revealed thinning of the pRNFL and PR layer in these patients compared to healthy controls. Based on the existing literature, these changes might constitute an early sign of retinal neurodegeneration, probably occurring as a result of an apoptotic process in the context of a chronic low-grade inflammatory microenvironment. Further research in this area should try to explore a possible relationship between the findings of this study and changes in structural and functional CNS imaging.

## Supplementary information

**Additional file 1: Table S1.** Pharmacological history of the patients by group.

**Additional file 2: Table S2.** Peripapillary retinal nerve fiber layer thickness (µm) in all seven sectors by group.

## Data Availability

The datasets used and/or analyzed during the current study are available from the corresponding author on reasonable request.
