# Supplementary material for: Neurodegeneration in systemic lupus erythematosus: layer by layer retinal study using optical coherence tomography
Source: Int J Retina Vitreous. 2020 Apr 21;6:15. doi: 10.1186/s40942-020-00219-y (PMC7171841; doi:10.1186/s40942-020-00219-y)
Supplement: Supplementary file 1 — Additional file 1: Table S1. Pharmacological history of the patients by group. [file 40942_2020_219_MOESM1_ESM.docx]

Table S1 Pharmacological history of the patients by group

| Variables | SLE group (n=68) | Control group (n=50) | p-value |
| --- | --- | --- | --- |
| Systemic steroids  Mean daily dose PDN equivalent (SD), mg | 31 (45.6)  10.1 (60.5) | 0  NA | -  - |
| Other immunosuppressives | 26 (38.2) | 0 | - |
| Biological agents | 7 (10.3) | 0 | - |
| ACE inhibitor | 10 (14.7) | 3 (6) | 0.136 |
| Angiotensin II receptor antagonist | 8 (11.8) | 3 (6) | 0.351 |
| Beta blocker | 8 (11.8) | 3 (6) | 0.351 |
| Diuretics | 4 (5.9) | 3 (6) | 1.000 |
| Calcium channel blocker | 6 (8.8) | 0 | 0.038 |
| Statins | 10 (14.7) | 17 (34) | 0.014 |
| Nitrates | 0 | 1 (2) | 0.424 |
| Antiplatelet therapy | 17 (25) | 1 (2) | 0.001 |
| Anticoagulant | 12 (17.6) | 0 | 0.002 |
| Thyroid hormones | 7 (10.3) | 1 (2) | 0.136 |
| Selective serotonin reuptake inhibitor | 10 (14.7) | 2 (4) | 0.057 |
| Tricyclic antidepressant | 3 (4.4) | 0 | 0.261 |
| Benzodiazepines | 8 (11.8) | 2 (4) | 0.187 |

The results are expressed as n (%). p-values were obtained by the chi-square or Fisher’s exact test, as appropriate.

Abbreviations: ACE, angiotensin-converting enzyme; NA, not applicable; PDN, prednisone; SLE, systemic lupus erythematosus.
