# Supplementary material for: Neurodegeneration in systemic lupus erythematosus: layer by layer retinal study using optical coherence tomography
Source: Int J Retina Vitreous. 2020 Apr 21;6:15. doi: 10.1186/s40942-020-00219-y (PMC7171841; doi:10.1186/s40942-020-00219-y)
Supplement: Supplementary file 2 — Additional file 2: Table S2. Peripapillary retinal nerve fiber layer thickness (µm) in all seven sectors by group. [file 40942_2020_219_MOESM2_ESM.docx]

Table S2 Peripapillary retinal nerve fiber layer thickness (µm) in all seven sectors by group

| Sectors | SLE group  (n = 68) | Control group  (n = 50) | p-value |
| --- | --- | --- | --- |
| Global | 96.59 (10.68) | 99.52 (7.29) | 0.097 |
| Temporal superior | 132.16 (15.81) | 140.10 (14.94) | 0.007 |
| Temporal | 68.00 (10.02) | 72.14 (11.23) | 0.037 |
| Temporal inferior | 141.85 (19.89) | 143.42 (18.56) | 0.664 |
| Nasal inferior | 113.93 (25.56) | 111.48 (20.80) | 0.580 |
| Nasal | 72.93 (13.23) | 74.00 (10.17) | 0.633 |
| Nasal superior | 104.35 (18.76) | 108.92 (21.68) | 0.224 |

The results are expressed as the mean (SD). p-values were obtained by univariable linear regression models.
